# Expression of Concern on “I*κ*B Kinase Inhibitor VII Modulates Sepsis-Induced Excessive Inflammation and Cardiac Dysfunction in 5/6 Nephrectomized Mice”

**DOI:** 10.1155/2023/9864040

**Published:** 2023-03-06

**Authors:** Mediators of Inflammation

*Mediators of Inflammation* would like to express concern with the article titled “I*κ*B Kinase Inhibitor VII Modulates Sepsis-Induced Excessive Inflammation and Cardiac Dysfunction in 5/6 Nephrectomized Mice” [1] due to concerns with the data as identified by the authors. The authors contacted the journal to request the retraction of the article stating that they were unable to repeat results shown in Figure 9. They explained that the manuscript states ‘When the 5/6 nephrectomized CLP mice were treated with IKK inhibitor VII, it caused a significant reduction in the increases seen in MPO activity and in levels of plasma inflammatory cytokines', however, when the experiments were repeated no significant difference was observed. During a reassessment of the article by the journal, potential concerns were identified in the Western blots of Figures 8 and 3, however, the resolution of the images prevented a conclusive result.

The authors apologized but did not provide a response to our request for further clarification and did not provide the original blots. As the journal has not received evidence that the findings are fundamentally flawed, it remains published, however, the conclusions should be viewed with caution.
